# Supplementary material for: The interactome of intact mitochondria by cross-linking mass spectrometry provides evidence for coexisting respiratory supercomplexes
Source: Mol Cell Proteomics. 2017 Dec 8;17(2):216–32. doi: 10.1074/mcp.RA117.000470 (PMC5795388; doi:10.1074/mcp.RA117.000470)
Supplement: Supplemental Data [file supp_RA117.000470_133922_0_supp_23978_fzffwf.zip › spectra_annotation/mito_DR_spectra_annotation/398-1-4-1-11-1.pdf]

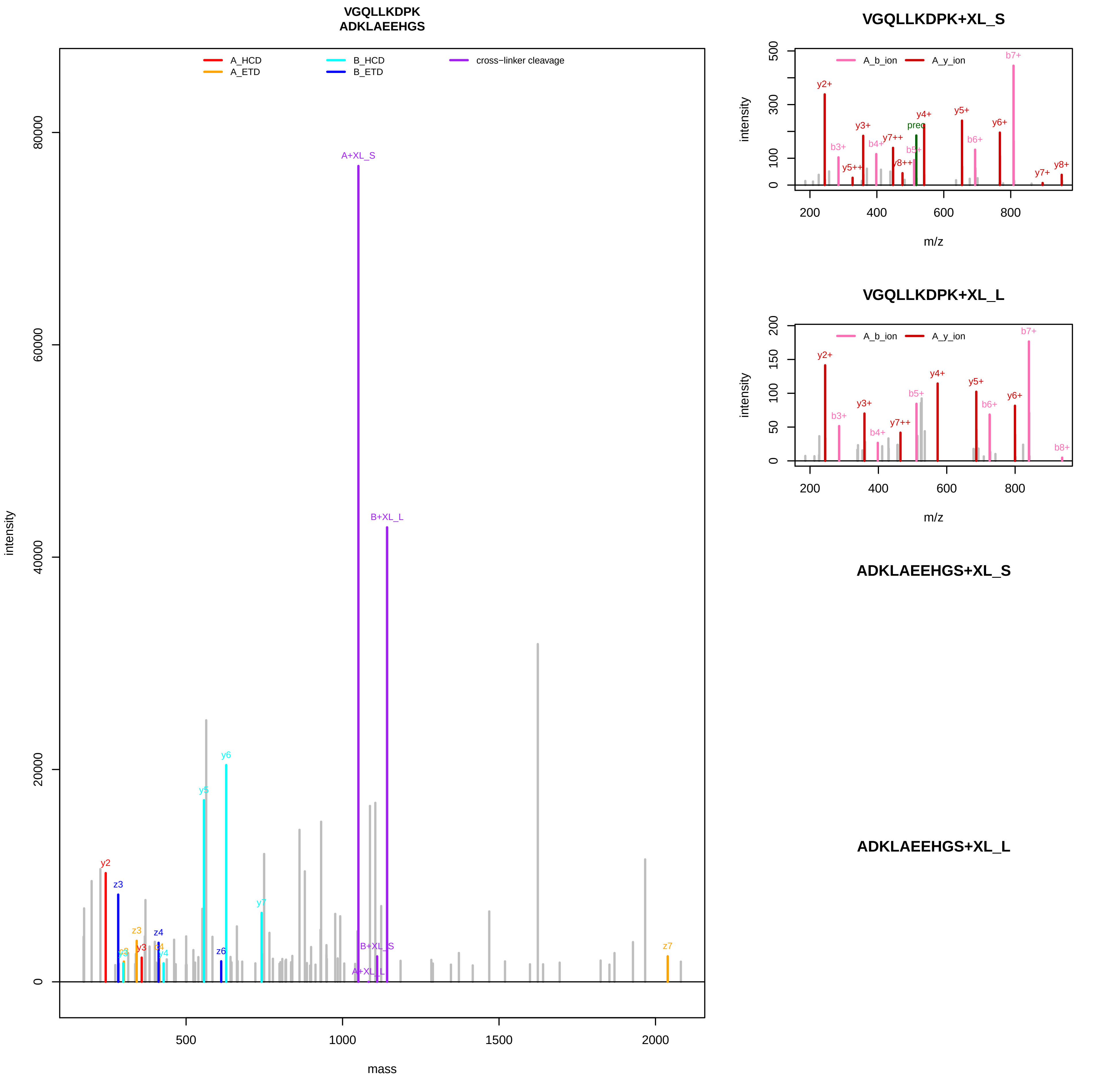
VGQLLKDPK
ADKLAEEHGS
A_HCD
A_ETD
B_HCD
B_ETD
cross−linker cleavage
A+XL_S
B+XL_L
B+XL_S
A+XL_L
y2
z3
z3
y3
z4
z6
y5
y6
y7
z7
intensity
mass
0
20000
40000
60000
80000
500
1000
1500
2000
VGQLLKDPK+XL_S
A_b_ion
A_y_ion
y2+
b3+
y5++
y3+
b4+
y7++
y8++
b5+
prec
y4+
y5+
b6+
y6+
b7+
y7+
y8+
intensity
m/z
0
100
300
500
200
400
600
800
VGQLLKDPK+XL_L
A_b_ion
A_y_ion
y2+
b3+
y3+
b4+
y7++
b5+
y4+
y5+
b6+
y6+
b7+
b8+
intensity
m/z
0
50
100
150
200
200
400
600
800
ADKLAEEHGS+XL_S
ADKLAEEHGS+XL_L
